# LesionQuant for Assessment of MRI in Multiple Sclerosis—A Promising Supplement to the Visual Scan Inspection

**DOI:** 10.3389/fneur.2020.546744

**Published:** 2020-12-11

**Authors:** Synne Brune, Einar A. Høgestøl, Vanja Cengija, Pål Berg-Hansen, Piotr Sowa, Gro O. Nygaard, Hanne F. Harbo, Mona K. Beyer

**Affiliations:** ^1^Institute of Clinical Medicine, University of Oslo, Oslo, Norway; ^2^Department of Neurology, Oslo University Hospital, Oslo, Norway; ^3^Division of Radiology and Nuclear Medicine, Oslo University Hospital, Oslo, Norway

**Keywords:** MRI, longitudinal lesions, brain atrophy, automatic lesion detection, multiple sclerosis

## Abstract

**Background and Goals:** Multiple sclerosis (MS) is a central nervous system inflammatory disease where magnetic resonance imaging (MRI) is an important tool for diagnosis and disease monitoring. Quantitative measurements of lesion volume, lesion count, distribution of lesions, and brain atrophy have a potentially significant value for evaluating disease progression. We hypothesize that utilizing software designed for evaluating MRI data in MS will provide more accurate and detailed analyses compared to the visual neuro-radiological evaluation.

**Methods:** A group of 56 MS patients (mean age 35 years, 70% females and 96% relapsing-remitting MS) was examined with brain MRI one and 5 years after diagnosis. The T1 and FLAIR brain MRI sequences for all patients were analyzed using the LesionQuant (LQ) software. These data were compared with data from structured visual evaluations of the MRI scans performed by neuro-radiologists, including assessments of atrophy, and lesion count. The data from LQ were also compared with data from other validated research methods for brain segmentation, including assessments of whole brain volume and lesion volume. Correlations with clinical tests like the timed 25-foot walk test (T25FT) were performed to explore additional value of LQ analyses.

**Results:** Lesion count assessments by LQ and by the neuro-radiologist were significantly correlated one year (cor = 0.92, *p* = 2.2 × 10^−16^) and 5 years (cor = 0.84, *p* = 2.7 × 10^−16^) after diagnosis. Analyzes of the intra- and interrater variability also correlated significantly (cor = 0.96, *p* < 0.001, cor = 0.97, *p* < 0.001). Significant positive correlation was found between lesion volume measured by LQ and by the software Cascade (cor = 0.7, *p* < 0.001. LQ detected a reduction in whole brain percentile >10 in 10 patients across the time-points, whereas the neuro-radiologist assessment identified six of these. The neuro-radiologist additionally identified five patients with increased atrophy in the follow-up period, all of them displayed decreasing low whole brain percentiles (median 11, range 8–28) in the LQ analysis. Significant positive correlation was identified between lesion volume measured by LQ and test performance on the T25FT both at 1 and 5 years after diagnosis.

**Conclusion:** For the number of MS lesions at both time-points, we demonstrated strong correlations between the assessments done by LQ and the neuro-radiologist. Lesion volume evaluated with LQ correlated with T25FT performance. LQ-analyses classified more patients to have brain atrophy than the visual neuro-radiological evaluation. In conclusion, LQ seems like a promising supplement to the evaluation performed by neuro-radiologists, providing an automated tool for evaluating lesions in MS patients and also detecting early signs of atrophy in both a longitudinal and cross-sectional setting.

## Introduction

Multiple sclerosis (MS) is a chronic inflammatory, demyelinating disorder of the central nervous system. Most frequently MS is characterized by a relapsing remitting disease course (RRMS) that over the years often converts to a secondary progressive disease course (SPMS). MS leads to variable degrees of physical and cognitive impairment. About 10 percent of the patients experience a progressive disease course from disease onset [primary progressive MS (PPMS)] (1, 2). A key challenge in MS care is to identify and develop prognostic biomarkers for the disease course (3). Magnetic resonance imaging (MRI) is still the most important tool for the diagnosis and monitoring of MS (4–6). MRI has a high sensitivity for the evaluation of inflammatory and neurodegenerative processes in the brain and spinal cord and it is the most commonly used method in the follow-up of MS patients (7).

Visual inspection of the MRI scans of people with MS is time consuming for the neuro-radiologist. Subjective measurements based on a radiologist's visual inspection may result in low reproducibility (8). In addition, the degrees of brain atrophy and MS-related pathology in the gray and white matter may be difficult to estimate, especially in early adulthood (9, 10).

Advanced tools have been shown necessary to detect early brain atrophy in MS (11). New MRI post-processing tools that automatically analyse complex brain volumetric information and lesion load have recently become commercially available. A study comparing two different software types for assessment of longitudinal whole brain atrophy in MS patients found a strong level of statistical agreement and consistency between the two programs in a real-world MS population (12). The authors conclude that automated measurements of atrophy show promise as biomarkers of neuro-degeneration in clinical practice and will enable more rapid clinical translation. If these types of programmes are to be introduced, in addition to showing their performance compared to competing programs or established research tools, we need to evaluate their use compared to clinical practice today. Do they perform as well, or better than current practice, and in what way can they be useful and valuable?

Although a neuro-radiological evaluation of structural brain MRI in MS patients can easily estimate the pathologic burden of abnormalities such as T2 hyperintense lesions, limited correlation exists between these measures and the clinical phenotype (13). This has been termed the “clinico-radiological paradox” and is well-described for both physical and cognitive impairments. Some explanations to this paradox have been suggested, including inappropriate clinical rating, and underestimation of damage to the normal appearing brain tissue (14). In a large meta-analysis including 2,891 patients, Mollison et al. found a modest correlation (*r* = –0.30) between MRI measures of total brain white matter lesions and cognitive function in people with MS (13).

LesionQuant(LQ) by CorTechs Labs is a software that automatically segments and measures volumes of brain structures and compares these volumes to norms based on the more established NeuroQuant(NQ) software (15). LQ was specifically designed for the evaluation of lesions and atrophy in MS patients. LQ also provides volumes and counts of new and enlarging brain lesions, and it automatically labels, visualizes and obtains the volumetric quantification of lesions based on brain MRI. LQ can therefore be used in the longitudinal follow-up of patients with MS. A recent study compared NQ to another software tool, Functional Magnetic Resonance Imaging of the Brain's (FMRIB's) Integrated Registration Segmentation Tool (FIRST), for estimating overall and regional brain volume in patients with clinically isolated syndrome (16). To our knowledge, no data is published that compares the LQ software in MS with visual evaluations performed by neuro-radiologists.

By using data from our prospective longitudinal study of newly diagnosed MS patients, results from longitudinal LQ analyses were compared to visual evaluations performed by neuro-radiologists in our hospital. We hypothesize that quantitative measurements of brain lesions and atrophy, using an unbiased automatic tool, may improve the correlation between clinical phenotype and MRI results.

Our aims were to evaluate (a) The performance of LQ at detecting brain lesions compared to a neuro-radiologist, and (b) brain atrophy as measured by LQ in comparison to the visual inspection by the neuro-radiologist. (c) The correlations of results from both visual assessment and LQ with clinically relevant variables and (d) the correlation between the segmented brain volumes and lesion volumes acquired from LQ and volumes from the two brain segmentation tools, FreeSurfer and Cascade.

## Materials and Methods

### Participants

All analyses were based upon carefully phenotyped MS patients in an ongoing prospective longitudinal MS study in Oslo (17, 18). A total of 56 MS patients were included in this study, which had been examined on average 1 year after diagnosis. The inclusion criteria were a diagnosis of RRMS in the period 2009–2012 and age 18–50 years. The exclusion criteria were a history of psychiatric or other neurological diseases than MS, drug abuse, previous adverse Gadolinium reaction, pregnancy or breast-feeding at inclusion, or non-fluency in Norwegian. Data from two time-points after diagnosis of MS were included in this study; data from time-point 1 (TP1) was collected 13 months after diagnosis (±9, *n* = 56) and data from time-point 2 (TP2) after 66 months (±12, *n* = 56). At both time-points an expanded disability status scale (EDSS) score was calculated based on a standard neurological examination by trained clinicians (19, 20). For assessment of walking ability and upper extremity function we also performed timed 25-foot walk test (T25FT) and 9-Hole Peg Test (9HPT). A brain MRI scan for clinical and research setting, was performed at both time-points.

We classified the disease modifying treatments (DMTs) as follows; group 0: no treatment, group 1: Glatiramer Acetate, Interferons, Teriflunomide or Dimetylfumarate, group 2: Fingolimod, Natalizumab or Alemtuzumab.

### MRI Acquisition

MS patients were scanned at both time-points with the same MRI scanning protocol in the same 1,5 T scanner (Avanto, Siemens Medical Solutions; Erlangen, Germany). The following two MRI sequences were required for the LQ analyses in this study;

(a) A Sagittal 3D T1 MPRAGE (FOV: 240 × 240 mm; slice thickness: 1.2 mm; voxel size: 1.3 × 1.3 × 1.2 mm; TR: 2,400 ms; TE: 3.61 ms; TI: 1,000 ms; flip angle: 8 deg.

(b) Pre-contrast sagittal 3D FLAIR (FOV: 260 × 260 mm; slice thickness: 1 mm; voxel size: 1 × 1 × 1 mm; TR: 6,000 ms; TE: 333 ms; TI: 2,200 ms.

The neuro-radiologist could in addition use all available other sequences in the study protocol, as mentioned in previous publications (17, 21).

### LesionQuant Analyses of Lesion Count, Brain Volume, and Lesion Volume

The MRI data from the 56 patients were analyzed using the LQ tool (version 2.3.0), comparing data at TP1 and TP2. For each patient, T1-weighted and FLAIR sequences were uploaded to the LQ server from the PACS system in the hospital, without the need for pre-processing. Finalized LQ-reports were received after ~10 min. The reports provided volumes and counts of all lesions, including new and enlarging lesions at TP2. A lesion was defined by LQ as being ≥ 4 mm^3^. Volumes of brain structures in the MS patients were compared with age and sex matched healthy controls from the LQ reference database. To establish the normative LQ database, CorTechs Labs combined data from several thousand scans including publicly available studies, studies by collaboration partners, and other proprietary data sources (age range 3–100 years, acquired using Siemens, GE and Philips MRI scanners with both 1.5T and 3T field strength).

**Figure 1:**
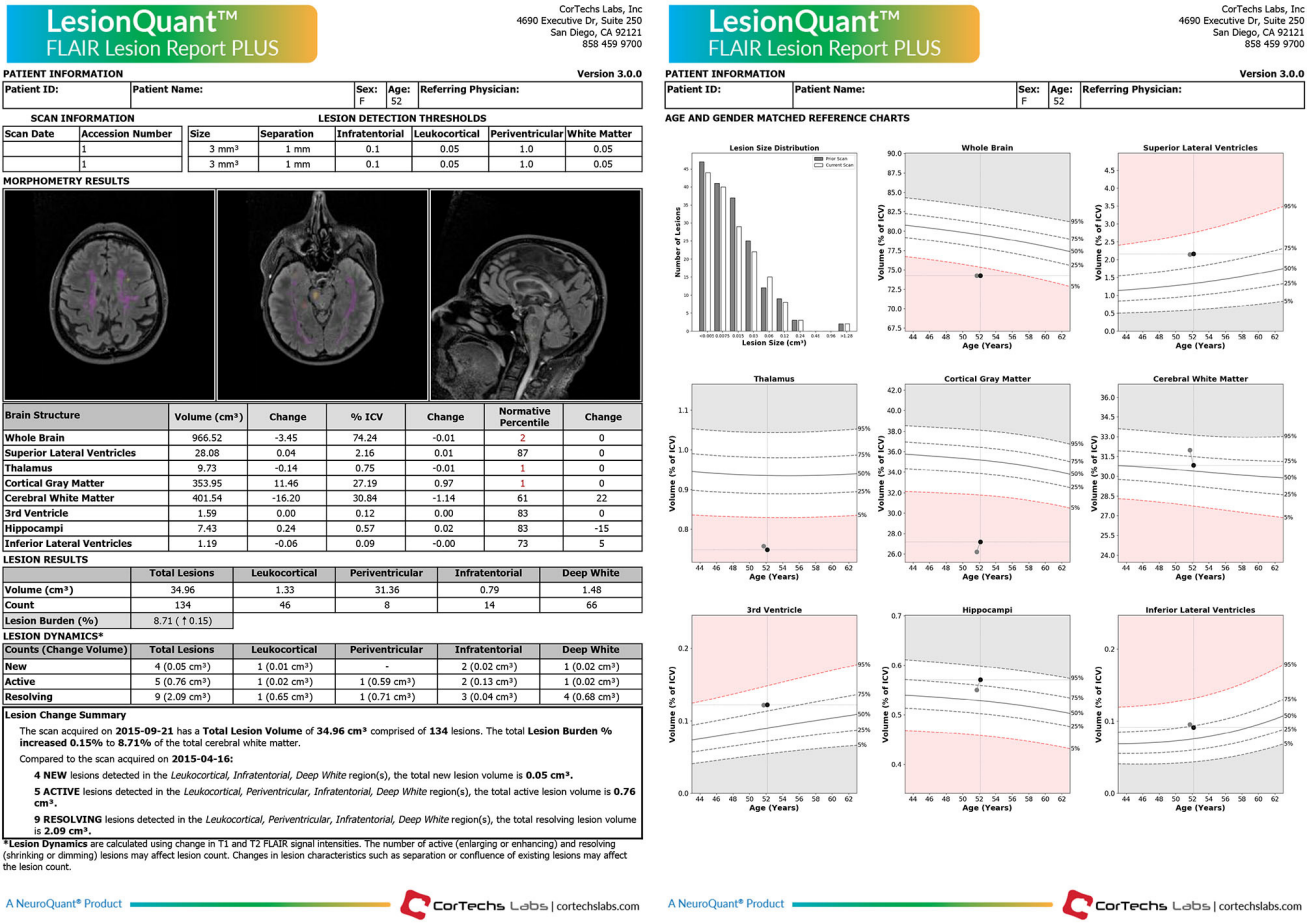
LesionQuant report. Example of a LesionQuant report from one MS subject comparing two MRI scans with a 5-month time interval between the two time-points.

The LQ-reports provided information about volumes of different brain structures, including whole brain, thalamus, cerebral white matter, volume of white matter lesions and cortical gray matter. The results for each patient are illustrated both using percentiles and absolute values (Figure 1). A cut off for atrophy was defined as a 10 percentile drop in brain volume for LQ between TP1 and TP2 (= 5-year interval).

### MRI Evaluation of Lesion Count and Brain Volume Presented by the Neuro-Radiologist

The brain MRIs from the included MS patients were systematically evaluated by a neuro-radiologist who carefully counted all lesions on the MRI scans of the patients at TP1 and TP2. A lesion was defined as having a high T2/FLAIR signal ≥ 3 millimeters in diameter. To evaluate the intrareader variability the neuro-radiologist read the data twice in 10 of the patients. A second neuro-radiologist also calculated the number of lesions in the same 10 patients to give information about the interrater variability. In addition to this, another method for evaluating the number of lesions was added. Two neuro-radiologists estimated a lesion number score at TP1. If the number of lesions were between 0 and 9 the lesion number score given was 1, if the number of lesions were between 10 and 19 the lesion number score given was 2, if the number of lesions were more than 20 the lesion number score given was 3.

**Figure 2:**
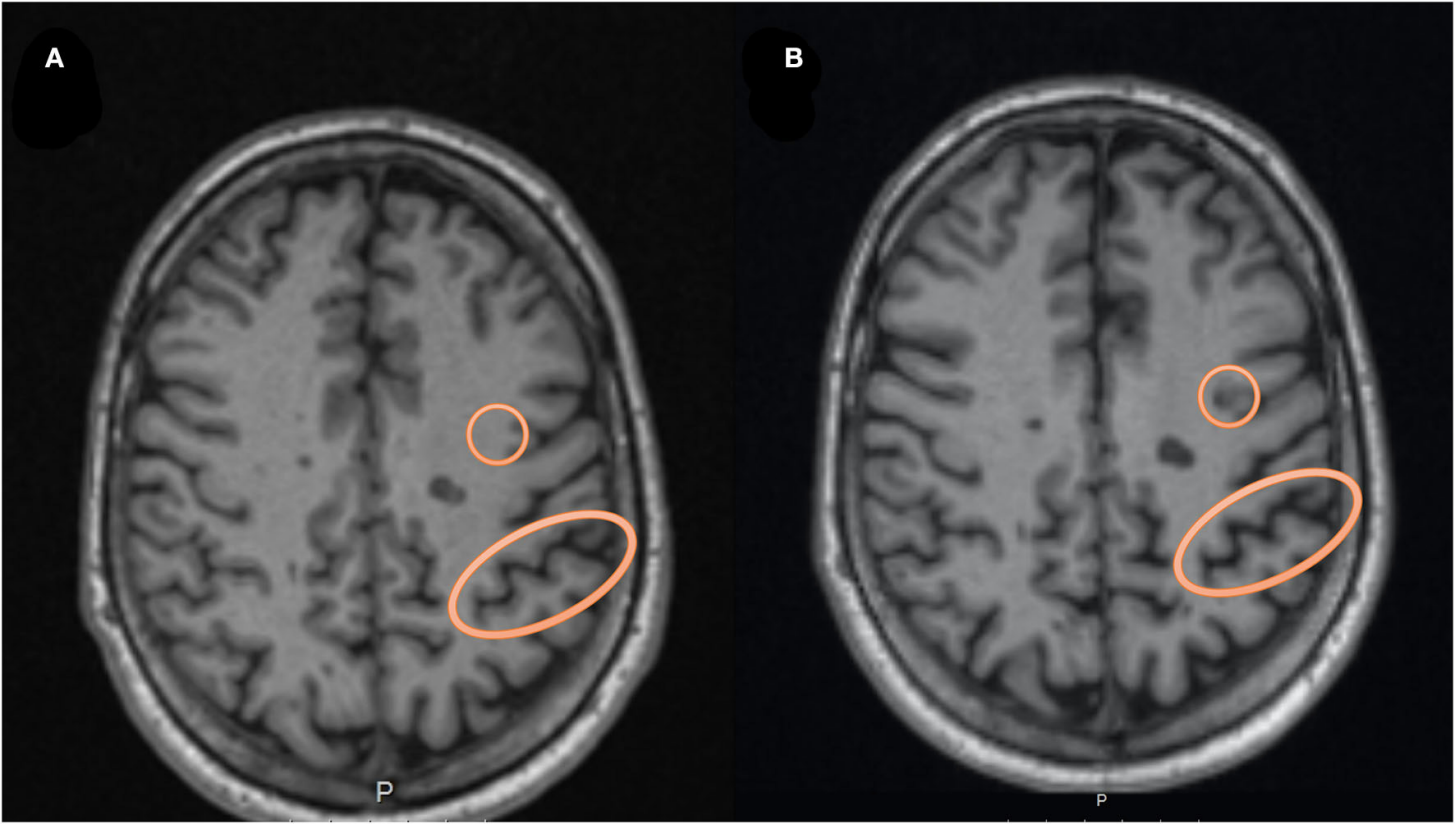
An example of the visual assessment by the neuro-radiologist. In **(A)** we see an axial T1 MRI at time-point 1, while in **(B)** we see the MRI at time-point 2, highlighting a circle with an example of a new lesion evolving during the follow-up period. The oval circle is an example of an area showing increased CSF in the sulcus, which was evaluated as representing atrophy between the two time-points.

A neuro-radiologist also assessed whether increased whole brain atrophy was visually evident between the two time-points or not (Figure 2). Visual evaluation of atrophy was done using the 3D T1 series, where increased CSF in the sulci or on the surface of the brain or volume loss of the gyri between TP1 and TP2, was regarded as atrophy.

The exact lesion number, lesion number score, and the evaluation of atrophy were then compared to the output from LQ. The assessment by the neuro-radiologist was used as the gold standard to compare the LQ data with.

### MRI Evaluation of Brain Volume and Lesion Volume Using Freesurfer and Cascade

To compare LQ with other research methods for brain segmentation we compared LQ with the softwares FreeSurfer and Cascade (22). To compare brain volume between the different softwares we used the measure for whole brain volume from both LQ (including brain stem) and FreeSurfer (excluding brain stem) (23). To compare the lesion volume between the different softwares we used the measure for total lesion volume from both LQ and Cascade.

### Statistical Analysis

We used R (R Core Team, Vienna, 2018, version 3.6.1) for statistical analyses. To assess reliability of the whole brain volumes from LQ and FreeSurfer we computed the intraclass correlation coefficient (ICC) using the R package “irr” (24). Figures were made using “ggplot2” (25) and “cowplot” (26) in R.

To evaluate the associations between the assessment provided by LQ, the neuro-radiologist, analysis using the FreeSurfer and Cascade softwares and the clinical data, we used the “stats” package in R and calculated the Pearson's correlation coefficient and applied the student's *t*-test (27).

To adjust for multiple comparisons, we calculated the degree of independence between the four clinical variables available, making a 4 × 4 correlation matrix based on the Pearson's correlation between all pair-wise combinations of clinical data. Utilizing the ratio of observed eigenvalue variance to its theoretical maximum, the estimated equivalent number of independent traits in our analyses was 3.0 (28). To control for multiple testing, our significance threshold was therefore adjusted accordingly from 0.05 to 0.017 (28).

## Results

### Participant Demographics and Characteristics

At TP1 mean age of the study participants was 36 years (range 21–49 years), 70% were females and 96% were classified as having RRMS. EDSS was stable between TP1 and TP2 with median EDSS 2.0 (range 0–6). Time since MS diagnosis was on average 12.9 months (SD = 9.3) at TP1 and 66.0 months (SD = 11.7) at TP2. At TP1, 25% did not receive any DMT for MS, 63% received a group 1 DMT (moderately effective treatment) and 12% a group 2 DMT (highly effective treatment). At TP2, 34% did not receive any DMT, 36% received a group 1 DMT and 30% a group 2 DMT (Table 1).

**Table 1:** Demographic and clinical characteristics of the multiple sclerosis patients.

	**Time-point 1**	**Time-point 2**
	***n=56***	***n=56***
**Demographic and clinical characteristics**		
Female, % (*n*)	70 (39)	70 (39)
Age, mean years (range)	35.8 (21–49)	40.3 (25–53)
Number of total attacks, mean (SD, range)	1.9 (1.0, 0–5)	2.6 (1.3)
Months since MS diagnosis, mean (SD, range)	12.9 (9.3, 0–34)	66.0 (11.7, 38–95)
**Clinical classification**		
EDSS, median (SD, range)	2.0 (1.0, 0–6)	2.0 (1.3, 0–6)
9-hole peg test, right hand, mean seconds (SD, range)	20.9 (3.4, 16.5–33.4)	21.3 (8.4, 15.3–73.7)
9-hole peg test, left hand, mean seconds (SD, range)	21.6 (3.0, 16.0–28.4)	21.1 (5.0, 16.6–45.5)
Timed 25-foot walk test, mean seconds (SD, range)	3.94 (0.75, 2.85–7.22)	4.05 (1.19, 2.49–9.49)
**Multiple sclerosis classification**		
RRMS, *n* (%)	54 (96)	53 (94)
PPMS, *n* (%)	1 (2)	1 (2)
SPMS, *n* (%)	1 (2)	2 (4)
**Disease modifying treatments**		
Group 0, *n* (%)	14 (25)	19 (34)
Group 1, *n* (%)	35 (63)	20 (36)
Group 2, *n* (%)	7 (12)	17 (30)

*EDSS, Expanded Disability Status Scale; Group 0, No Treatment; Group 1, Glatiramer Acetate, Interferons, Teriflunomide or Dimetylfumarate; Group 2, Fingolimod, Natalizumab or Alemtuzumab; PPMS, Primary progressive; MS, RRMS, Relapsing-remitting MS, SD, Standard Deviation; SPMS, Secondary progressive MS*.

### Cross-Sectional Comparison of Lesion Count Between the LesionQuant Reports and the Neuro-Radiological Evaluations

The lesion count assessments by LQ and the neuro-radiologist were significantly correlated at TP1 (cor = 0.92, *p* = 2.2 × 10^−16^) and TP2 (cor = 0.84, *p* = 2.7 × 10^−16^) (Supplementary Figure 2). The lesion counts were identical in only two and three patients at TP1 and TP2, respectively. While lesion counts were higher by the neuro-radiologist in 39 and 40 patients at TP1 and TP2, respectively. Lesion counts were lower by LQ in 15 and 13 patients at TP1 and TP2, respectively. In general, the differences in number of lesions evaluated by LQ and the neuro-radiologist increased with age. For patients with higher number of lesions the neuro-radiologist tended to count more lesions than LQ, and the opposite with lower number of lesions, see Supplementary Figure 1. To evaluate the intra and interrater variability the neuro-radiologist recounted the lesions in 10 of the patients, and a second neuro-radiologist counted lesions in the same 10 patients. Both the intra- and the inter-rater variability were significantly correlated (cor = 0.96, *p* < 0.001, cor = 0.97, *p* < 0.001). This was also the case for the lesion number scores estimated between LQ, and two different neuro-radiologists.

The lesion volume assessments by LQ and the Cascade software were significantly correlated (cor = 0.7, *p* < 0.001).

### Longitudinal Evaluations of Atrophy and Lesions

We also compared the LQ software with the assessment by the neuro-radiologist in identifying whole brain atrophy at TP2. The neuro-radiologist classified 12 subjects to have brain atrophy. These 12 subjects also had significantly lower scores on whole brain atrophy by LQ (mean brain volume percentile 37.0, range 10–80), compared to the subjects that were not classified as having brain atrophy (mean brain volume percentile 48.7, range 2–99).

At TP2, LQ and the neuro-radiologist agreed in classifying 33% of the subjects with atrophy (four out of 12 subjects). In addition, the neuro-radiologist identified eight more subjects with brain atrophy (mean LQ whole brain percentile 31.3).

LQ detected a reduction in whole brain percentile >10 in 10 patients between TP1 and TP2, while the neuro-radiological evaluation identified six of these. The evaluation by the neuro-radiologist identified an additional six patients with increased atrophy between TP1 and TP2, all of whom displayed low whole brain percentiles at TP2 (median 11, range 8–28) and decreasing percentile between the time-points.

At TP2 we found that LQ showed reduced whole brain volume in 51 patients compared to TP1 with a mean reduction in volume of 20.5 ml/1.59% (range 0.4–109.4 ml/0.03–8.08%) of the whole brain volume. In the remaining five patients we found an increased volume with a mean increase in volume of 6.8 ml/ 0.56% (range 0.2–17.4 ml/0.02–1.44%).

**Figure 3:**
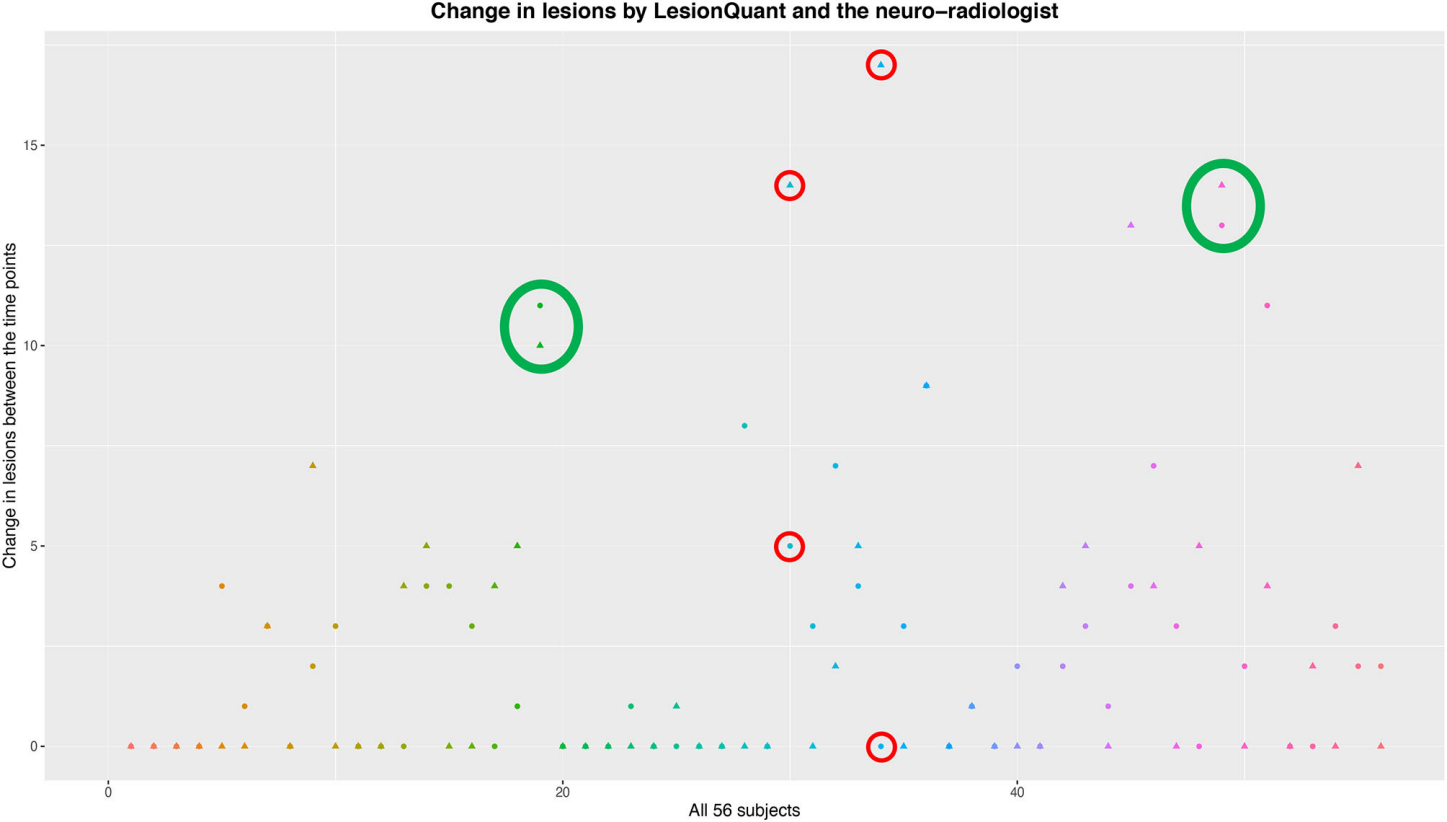
An overview of the evaluations of change in lesions between the two time-points. The LesionQuant assessments are depicted with a circle, while the neuro-radiological evaluations are depicted using a triangle. Each subject is visualized with both assessments and with a unique color. The green circles show examples of assessments with good agreement between LQ and neuro-radiologist, while the red circles show assessments where the two methods differ a lot in the same patient.

To evaluate the sensitivity of LQ in detecting new lesions, compared to the neuro-radiologist, the difference in number of lesions assessed at the two time-points was analyzed in a 2 × 2 table (Table 2). The sensitivity of the LQ-analysis to correctly classify the patients according to the gold standard neuro-radiological evaluation was 53% (17/32 patients). The specificity of the LQ-analysis to correctly evaluate the MRI follow-up as stable according to the neuro-radiological evaluation was 75% (18/24). In total, 43 % of the patients were evaluated with no new lesions on MRI at TP2 by the neuro-radiologist. Also, 57% (32 patients) had new lesions according to the neuro-radiologist, and only 17 of these had new lesions according to the LQ-reports (Table 2 and Figure 3).

**Table 2:** A 2 × 2 table based on the ability to capture MRI activity based on the presence of new lesions in our longitudinal MS sample.

		**LesionQuant report**		
		**New lesions**	**Stable**	**In total**
Neuro-radiological evaluation	New lesions	17	15	32
	Stable	6	18	24
	In total	23	33	56

### Correlations Between MRI Features and Clinical Variables

We found significant positive correlations between T25FT and the lesion volume as measured by LQ at both TP1 (*t* = 3.08, *p* = 3.2 × 10^−3^) and TP2 (*t* = 3.72, *p* = 4.8 × 10^−4^) (Table 3). These results also indicate slower test performance by T25FT in patients with a higher burden of lesion volume. In addition, we found a significant positive correlation between the 9HPT using the left hand and lesion volume at TP2 (*t* = 5.34, *p* = 2.09 × 10^−6^), indicating slower test performance with increased lesion volume. We also found a significant negative correlation with EDSS and whole brain volume at TP1 (*t* = −2.68, *p* = 9.8 × 10^−3^), indicating higher EDSS scores with lower brain volumes. We found no significant correlations between the number of lesions reported by the neuro-radiologist and the clinical variables. All significant correlations reported were adjusted for multiple testing.

**Table 3:** Associations between LQ-variables and clinical variables.

	**LesionQuant variables**			
	**Lesion volume**			
	**Time-point 1**		**Time-point 2**	
**Clinical variables**	*t*-value	*p*-value	*t*-value	*p*-value
Expanded disability status scale	1.12	0.27	1.43	0.16
9-hole peg test, right hand	2.00	0.05	1.87	0.07
9-hole peg test, left hand	1.00	0.32	***5.34***	***2.09 x 10**^**−6**^*
Timed 25-foot walk test	***3.08***	***3.2 x 10**^**−3**^*	***3.72***	***4.8 x 10**^**−4**^*
	**Lesion count**			
Expanded disability status scale	0.85	0.40	0.42	0.68
9-hole peg test, right hand	2.35	0.02	0.50	0.62
9-hole peg test, left hand	−0.23	0.82	1.90	0.06
Timed 25-foot walk test	1.48	0.15	0.68	0.50
	**Whole brain volume**			
Expanded disability status scale	***−2.68***	***9.8 x 10**^**−3**^*	−1.45	0.15
9-hole peg test, right hand	0.94	0.35	0.86	0.40
9-hole peg test, left hand	−2.24	0.03	−0.20	0.84
Timed 25-foot walk test	−0.68	0.50	−1.22	0.23
	**Whole brain percentile**			
Expanded disability status scale	−0.11	0.91	−0.81	0.42
9-hole peg test, right hand	1.05	0.30	−0.33	0.74
9-hole peg test, left hand	−1.16	0.25	−0.69	0.49
Timed 25-foot walk test	1.06	0.29	0.18	0.86
	**Lesion count by neuro-radiologist**			
Expanded disability status scale	1.00	0.33	1.06	0.30
9-hole peg test, right hand	2.64	0.01	0.76	0.45
9-hole peg test, left hand	0.05	0.96	2.69	0.01
Timed 25-foot walk test	1.11	0.27	1.20	0.24

*Results marked in bold and italics were significant after adjusting for multiple testing*.

### Reliability of LesionQuant Volumes

To validate the LQ data with the established FreeSurfer output for brain segmentation we compared the measure for whole brain volume from both LQ (including brainstem) and FreeSurfer (excluding brain stem), (Supplementary Figure 2). At both TP1 (*t* = 51.6, cor = 0.99) and TP2 (*t* = 45.2, cor = 0.99), Pearson's correlations were highly significant. We also validated regional volumes for thalamus using both raw FreeSurfer data and data processed through the longitudinal stream compared to the LQ data, yielding less significant correlations (*t* = 2.4–2.8 and, cor = 0.32–0.35, *p* = 0.02–0.008).

### LesionQuant Reports and Neuro-Radiological Evaluation

All longitudinal LQ-data yielded excellent concurrence. To evaluate the consistency and agreement of the longitudinal LQ-reports, we measured the Intraclass correlation coefficients (ICC) between TP1 and TP2 for brain volume (ICC = 0.97, *p* = 2 × 10^−35^), lesion count (ICC = 0.91, *p* = 1.4 × 10^−23^), lesion volume (ICC = 0.88, *p* = 5.2 × 10^−20^) and thalamus volume (ICC = 0.91, *p* = 3.0 × 10^−23^) (Supplementary Table 1). We found significant correlations between lesion volume and the number of lesions at both TP1 (*t* = 6.32, *p* = 5.05 × 10^−8^) and TP2 (*t* = 4.21, *p* = 9.77 × 10^−5^). We found no significant changes in the parameters between TP1 and TP2 (Supplementary Table 1). As a sanity check, the ICC for lesion counts reported by the neuro-radiologist was very high (ICC = 0.99, *p* = 2.6 × 10^−50^), as expected.

## Discussion

Magnetic resonance imaging is an important para-clinical tool for the diagnosis and monitoring of MS. Quantitative measurements of lesion volume and distribution of lesions have a significant value for evaluating disease progression in a clinical setting and brain atrophy is a possible new measurement to be used in future evaluation in MS patients. In this study we explored the use of the LQ software for evaluating cerebral MS lesions as well as brain atrophy in a clinical setting, and investigated if an automatic analysis of MRI scans using such software shows promise for use in the clinical follow-up of MS-patients.

We found a high correlation between lesions counted by the neuro-radiologist at TP1 and TP2 and the lesion count output from LQ. In evaluation of atrophy between TP1 and TP2 there was only agreement between the neuro-radiologist and LQ in 50% of the patients (6 out twelve). Differences in whole brain percentiles between TP1 to TP2 were detected with LQ in the majority of patients, ranging between 0.03 and 8.08%. Lesion volume from LQ analysis correlated with outcome of clinical tests of walking speed and upper extremity function. Significant positive correlation was identified between lesion volume measured by LQ and test performance on the T25FT both at 1–5 years after diagnosis. There was a significant correlation between the results of LQ and the segmented volumes by FreeSurfer, showing high reliability of LQ output for whole brain volume. The correlation between lesion volume estimated by LQ and by the Cascade software were also highly significant.

In order to evaluate treatment-effect, it is of importance to see if new or enlarging lesions appear on a follow-up MRI scan. The lesion counts of the LQ software and the neuro-radiologist were highly correlated at both timepoints. However, visual assessment revealed somewhat higher lesion counts than the LQ assessment, more so in patients with a high number of lesions. The explanation of this difference in lesion number could be explained in differences in the definition of a lesion. As mentioned in the materials and method chapter a lesion was defined as having a T2/FLAIR signal ≥ 3 millimeters in diameter when analyzed by visual evaluation, but the lesion size set by LQ was ≥ 4 mm^3^. With the high correlation of lesion count overall, the LQ tool should be valuable for detecting lesions in routine follow-up MRI in MS. The resulting LQ report could then be controlled by a neuro-radiologist.

Regarding the detection of lesions, we used the assessment by the neuro-radiologist as the “gold standard.” However, it is well-established that the detection of cortical lesions may be challenging using conventional brain MRI and these may be missed by radiologists (29). This is shown in a study comparing the number of MS lesions counted by radiologists and as analyzed by MSmetrix (today known as IcoBrain MS), a software comparable to LQ (30). This study showed a higher recount-difference when recounting was performed by radiologists than in MSmetrix (31). Therefore, the gold standard as we defined it in this paper, may be more variable than the automated software tool.

Reliable evaluation of atrophy is difficult with only visual inspection, although it is not a very time-consuming task. Results from studies comparing visual ratings of atrophy using GCA have shown Inter-rater reliability of > 0.6 and Intra-rater reliability of >0.7, which is considered moderate agreement (32). When the neuro-radiologist evaluated the MRI, images from TP2 was compared with the MRI scan at TP1 for each patient. In a clinical routine setting, the neuro-radiologists often compare to the previous MRI, which may be taken months or up to a year before. The changes in atrophy are rather small from year to year −0.2 to −0.3% per year in our patients' age range) (33) and it is not possible to detect such small changes in reduction of brain volume for the neuro-radiologist. A better approach may be to always compare the last scan to the first MRI in order to increase sensitivity of visual atrophy assessment. But even if there are several years between the MRI scans it could be difficult to estimate reduction in brain volume if the patients evaluated are young and stable. The discrepancy between the 12 patients found to have atrophy from visual inspection, to the 51 patients showing reduced brain volumes in the 5-year follow-up may indicate that LQ would be helpful in clinical practice. Never the less we should have in mind the risk of detecting reductions in whole brain volume with LQ which is not clinically relevant. Our MS population is young (mean age 36 years) and relatively stable (median EDSS 2,0). Finding small reductions in brain volume in such a population would not necessarily improve their general health condition. But overall, our results indicate that the automated method LQ performs better than the visual evaluation method in terms of atrophy evaluations, as discussed above.

Most of the MS patients were treated with moderately or highly efficacious disease modifying therapies at TP1 and TP2. In total, 10 MS patients changed to a more efficient MS treatment during the follow-up. We found no significant differences in brain volumes or change in brain volumes between the patients who increased treatment efficiency during the follow-up and those who either used the same treatment or reduced the efficacy of their MS treatment during the follow-up period. As a confounding factor we have to consider that switching to more efficacious treatments would impact the brain volumes by possible pseudoatrophy during the first 6 months (34). Although, during our observational period we did not find any significant differences. Other short term confounding factors affecting brain volume measurements are known, such as fluid restriction, the time of the day for MRI measurements, corticosteroids, antipsychotic treatment and short-term effects of pathological processes that decrease global brain volume (35).

LQ compared differences in brain volume during approximately a 5-year period (2012/2013 and 2016/2017). During this period the patients were scanned on several occasions, which were not part of the study. One of the main benefits of using automated methods for image analysis in MS patients, is the possibility to perform more reliable and quick evaluation of brain atrophy. As shown by Pareto et al. when comparing two different tools for automated volume analysis of different brain regions, the size of the brain region of interest seems to be important (16). We found an excellent correlation between the FreeSurfer and LQ software's in the assessment of whole brain atrophy (cor = 0.99). However, for thalamus we only found modest correlations between both the raw and processed volumes estimated by LQ and FreeSurfer (cor = 0.32–0.35, *p* = 0.02–0.008, respectively), confirming the results of Pareto et al. In a recent paper, Storelli et al. also studied reproducibility and repeatability using different software's for atrophy measurements (10). They concluded that an improved reproducibility between scanners is required for clinical application.

In our study the LQ software estimated an unexpected increased whole brain volume in six patients between TP1 and TP2. This could be due to variability in the MRI scanner or other technical reasons. Alternatively, changes in lesion load in the patient over time may affect the atrophy measurements (10).

We hypothesized that improved measurements of brain lesions and atrophy, using an unbiased automatic tool, may improve the correlation between clinical phenotype and MRI results. We found that only the automated LQ software was able to show significant correlation with the standard clinical tests (T25FT, 9HPT, and EDSS). We consider this to be a robust and expected finding as only LQ and not the neuro-radiologist could provide volumetric information. In line with this, the 9HPT was positively correlated with lesion volume at time-point two; although only significant for the left hand, the same trend was seen for the right hand. The EDSS scale, which is the most widely used method to grade disability in MS, was only associated with whole brain volume at TP1 (*t* = −2.68, *p* = 9.8 × 10^−3^). There were no correlations between lesion count, either from LQ or the neuro-radiologist and the EDSS, T25FT or 9HPT, also showing the value of having volumes of the lesions and whole brain available.

In general, we found very high levels of intraclass correlation coefficients (0.88–0.97), showing consistency and agreement among the longitudinal LQ-reports. A strength of this paper is the longitudinal design where the MS patients were examined clinically and with brain MRI both one and 5 years after diagnosis. The patient cohort is well-characterized by trained clinicians, performing the clinical and MRI assessments. The same MRI scanner and protocol was used for all patients at the two time-points of evaluation, and all patients were scanned in the afternoon/early evening. The neuro-radiological evaluation of the 56 patients at TP1 and TP2 was performed by the same neuro-radiologist, and in addition both inter and intrarater evaluations were performed. Thus, the quality of the data included in this study is suitable for addressing the research question. A weakness of the study is that we did not perform visual assessment by two independent raters for the visual evaluation of atrophy. Also, there was no control group.

The structured LQ report is acquired using fully automated MRI post-processing software, which requires only minimal effort and reduces bias of different raters, which is present when using visual inspection of images. Another advantage is the very short processing time of LQ compared to similar software used for research, with only about 10 min from the uploading of images to the final report is received. In comparison, software like FreeSurfer needs hours to process the data, cannot be interpreted for individual patients and is not feasible for clinical practice.

## Conclusion

In conclusion, automatic analyses of MRI scans of MS patients may provide faster assessments than the traditional evaluation performed by the neuro-radiologist. LQ seems like a promising supplement to the evaluation by the neuro-radiologist, providing an automated tool for assessment of MS lesions and brain volume in MS patients.

## Data Availability Statement

The current dataset cannot be made publicly available for ethical reasons, and public availability would compromise patient confidentiality and participant privacy. The study was conducted in humans and the dataset includes sensitive and personal information on individuals. A portion of data can be made available upon request to interested, qualified researchers provided that an agreement is made up. The minimal data set will enable replication of the reported study findings. Requests to access the datasets should be directed to Hanne F. Harbo, h.f.harbo@medisin.uio.no.

## Ethics Statement

The studies involving human participants were reviewed and approved by The South Eastern Regional Committee for Medical and Health Research Ethics. The patients/participants provided their written informed consent to participate in this study.

## Author Contributions

SB, EH, PB-H, HH, and MB contributed to the conception, design of the study, and drafted the text and figures. SB, EH, VC, GN, PB-H, HH, PS, and MB contributed to the acquisition and analysis of data. During review and editing of this manuscript, all authors contributed.

## Conflict of Interest

SB has received honoraria for lecturing from Biogen and Novartis. EH has received honoraria for lecturing from Biogen, Merck and Sanofi-Genzyme. PB-H has received advisory board and/or speaker honoraria from Biogen, Novartis, Merck, UCB, and Teva. PS has received honoraria for lecturing and travel support from Merck. HH has received travel support, honoraria for advice or lecturing from Biogen Idec, Sanofi-Genzyme, Merck, Novartis, Roche, and Teva and an unrestricted research grant from Novartis and Biogen. MB has received honoraria for lecturing from Novartis and Biogen Idec, Merck AB, Roche Norge, and Sanofi Genzyme. The remaining authors declare that the research was conducted in the absence of any commercial or financial relationships that could be construed as a potential conflict of interest.

## References

[1] Koch M Kingwell E Rieckmann P Tremlett H The natural history of primary progressive multiple sclerosis. Neurology. (2009) 73:1996–2002. 10.1212/WNL.0b013e3181c5b47f 19996074

[2] Lublin FD Reingold SC Cohen JA Cutter GR Sørensen PS Thompson AJ Defining the clinical course of multiple sclerosis: the 2013 revisions. Neurology. (2014) 83:278–86. 10.1212/WNL.0000000000000560 24871874 PMC4117366

[3] Katsavos S Anagnostouli M Biomarkers in multiple sclerosis: an up-to-date overview. Mult Scler Int. (2013) 2013:340508 10.1155/2013/340508 23401777 PMC3564381

[4] Wattjes MP Rovira À Miller D Yousry TA Sormani MP de Stefano MP. Evidence-based guidelines: MAGNIMS consensus guidelines on the use of MRI in multiple sclerosis–establishing disease prognosis and monitoring patients. Nat Rev Neurol. (2015) 11:597–606. 10.1038/nrneurol.2015.157 26369511

[5] Filippi M Rocca MA Ciccarelli O De Stefano N Evangelou N Kappos L. MRI criteria for the diagnosis of multiple sclerosis: MAGNIMS consensus guidelines. Lancet Neurol. (2016) 15:292–303. 10.1016/S1474-4422(15)00393-2 26822746 PMC4760851

[6] Geraldes R Ciccarelli O Barkhof F De Stefano N Enzinger C Filippi M The current role of MRI in differentiating multiple sclerosis from its imaging mimics. Nat Rev Neurol. (2018) 14:213 10.1038/nrneurol.2018.39 29582852

[7] Kaunzner UW Gauthier SA. MRI in the assessment and monitoring of multiple sclerosis: an update on best practice. Ther Adv Neurol Disord. (2017) 10:247–61. 10.1177/1756285617708911 28607577 PMC5453402

[8] Dwyer MG Silva D Bergsland N Horakova D Ramasamy D Durfee J. Neurological software tool for reliable atrophy measurement (NeuroSTREAM) of the lateral ventricles on clinical-quality T2-FLAIR MRI scans in multiple sclerosis. NeuroImage Clin. (2017) 15:769–79. 10.1016/j.nicl.2017.06.022 28706852 PMC5496213

[9] Eshaghi A Marinescu RV Young AL Firth NC Prados F Jorge Cardoso M. Progression of regional grey matter atrophy in multiple sclerosis. Brain. (2018) 141:1665–77. 10.1093/brain/awy088 29741648 PMC5995197

[10] Storelli L Rocca MA Pagani E Van Hecke W Horsfield MA De Stefano N. Measurement of whole-brain and gray matter atrophy in multiple sclerosis: assessment with MR imaging. Radiology. (2018) 288:554–64. 10.1148/radiol.2018172468 29714673

[11] Filippi M. MRI measures of neurodegeneration in multiple sclerosis: implications for disability, disease monitoring, and treatment. J Neurol. (2015) 262:1–6. 10.1007/s00415-014-7340-9 24723117

[12] Beadnall HN Wang C Van Hecke W Ribbens A Billiet T Barnett MH. Comparing longitudinal brain atrophy measurement techniques in a real-world multiple sclerosis clinical practice cohort: towards clinical integration? Ther Adv Neurol Disord. (2019) 12:1756286418823462. 10.1177/1756286418823462 30719080 PMC6348578

[13] Mollison D Sellar R Bastin M Mollison D Chandran S Wardlaw J. The clinico-radiological paradox of cognitive function and MRI burden of white matter lesions in people with multiple sclerosis: a systematic review and meta-analysis. PLoS ONE. (2017) 12:e0177727. 10.1371/journal.pone.0177727 28505177 PMC5432109

[14] Barkhof F. The clinico-radiological paradox in multiple sclerosis revisited. Curr Opin Neurol. (2002) 15:239–45. 10.1097/00019052-200206000-00003 12045719

[15] Brewer JB. Fully-automated volumetric MRI with normative ranges: translation to clinical practice. Behav Neurol. (2009) 21:21–8. 10.1155/2009/616581 19847042 PMC5444284

[16] Pareto D Sastre-Garriga J Alberich M Auger C Tintoré M Montalban X. Brain regional volume estimations with NeuroQuant and FIRST: a study in patients with a clinically isolated syndrome. Neuroradiology. (2019) 61:667–74. 10.1007/s00234-019-02191-3 30834955

[17] Nygaard GO Celius EG de Rodez Benavent SA Sowa P Gustavsen MW Fjell AM. A Longitudinal study of disability, cognition and gray matter atrophy in early multiple sclerosis patients according to evidence of disease activity. PLoS ONE. (2015) 10:e0135974. 10.1371/journal.pone.0135974 26280173 PMC4539191

[18] Høgestøl EA Kaufmann T Nygaard GO Beyer MK Sowa P Nordvik JE. Cross-sectional and longitudinal mri brain scans reveal accelerated brain aging in multiple sclerosis. Front Neurol. (2019) 10:450. 10.3389/fneur.2019.00450 31114541 PMC6503038

[19] Kurtzke JF. Rating neurologic impairment in multiple sclerosis: an expanded disability status scale (EDSS). Neurology. (1983) 33:1444–52. 10.1212/WNL.33.11.1444 6685237

[20] Cutter GR Baier ML Rudick RA Cookfair DL Fischer JS Petkau J. Development of a multiple sclerosis functional composite as a clinical trial outcome measure. Brain. (1999) 122 (Pt 5):871–82. 10.1093/brain/122.5.871 10355672

[21] Nygaard GO Walhovd KB Sowa P Chepkoech JL Bjørnerud A Due-Tønnessen P. Cortical thickness and surface area relate to specific symptoms in early relapsing-remitting multiple sclerosis. Mult Scler. (2015) 21:402–14. 10.1177/1352458514543811 25139946

[22] Damangir S Manzouri A Oppedal K Carlsson S Firbank MJ Sonnesyn H. Multispectral MRI segmentation of age related white matter changes using a cascade of support vector machines. J Neurol Sci. (2012) 322:211–6. 10.1016/j.jns.2012.07.064 22921728

[23] Dale AM Fischl B Sereno MI. Cortical surface-based analysis. I. Segmentation and surface reconstruction. Neuroimage. (1999) 9:179–94. 9931268 10.1006/nimg.1998.0395

[24] Gamer MLJ Singh. Various Coefficients of Interrater Reliability and Agreement. (2019).

[25] Wickham H ggplot2: Elegant Graphics for Data Analysis. 2 ed: Springer International Publishing (2016).

[26] Wilke C cowplot: Streamlined Plot Theme and Plot Annotations for 'ggplot2' 2019. [Available online at: https://wilkelab.org/cowplot/].

[27] RCTTRSP The R Stats Package. Available online at: https://stat.ethz.ch/R-manual/R-devel/library/stats/html/00Index.html~2019

[28] Li J Ji L. Adjusting multiple testing in multilocus analyses using the eigenvalues of a correlation matrix. Heredity. (2005) 95:221–7. 10.1038/sj.hdy.6800717 16077740

[29] Patel KR Luo J Alvarez E Piccio L Schmidt RE Yablonskiy DA. Detection of cortical lesions in multiple sclerosis: a new imaging approach. Mult Scler J Exp Transl Clin. (2015) 1:2055217315606465. 10.1177/2F2055217315606465 28607704 PMC5433400

[30] Jain S Sima DM Ribbens A Cambron M Maertens A Van Hecke W. Automatic segmentation and volumetry of multiple sclerosis brain lesions from MR images. NeuroImage Clin. (2015) 8:367–75. 10.1016/j.nicl.2015.05.003 26106562 PMC4474324

[31] Sima FPD Torcida GN Wilms A Lysandropoulos WVH (editors). Impact of MSmetrix Automatic Lesion Segmentation on the 32 Visual Count of Multiple Sclerosis Lesions. ECR (2018).

[32] Pasquier F Leys D Weerts JG Mounier-Vehier F Barkhof F Scheltens P. Inter- and intraobserver reproducibility of cerebral atrophy assessment on MRI scans with hemispheric infarcts. Eur Neurol. (1996) 36:268–72. 10.1159/000117270 8864706

[33] Battaglini M Gentile G Luchetti L Giorgio A Vrenken H Barkhof F. Lifespan normative data on rates of brain volume changes. Neurobiol Aging. (2019) 81:30–7. 10.1016/j.neurobiolaging.2019.05.010 31207467

[34] De Stefano N Arnold DL. Towards a better understanding of pseudoatrophy in the brain of multiple sclerosis patients. Mult Scler. (2015) 21:675–6. 10.1177/1352458514564494 25623248

[35] Dieleman N Koek HL Hendrikse J. Short-term mechanisms influencing volumetric brain dynamics. NeuroImage Clin. (2017) 16:507–13. 10.1016/j.nicl.2017.09.002 28971004 PMC5609861

