# Supplementary figures and images for: LesionQuant for Assessment of MRI in Multiple Sclerosis—A Promising Supplement to the Visual Scan Inspection
Source: Front Neurol. 2020 Dec 11;11:546744. doi: 10.3389/fneur.2020.546744 (PMC7759639; doi:10.3389/fneur.2020.546744)

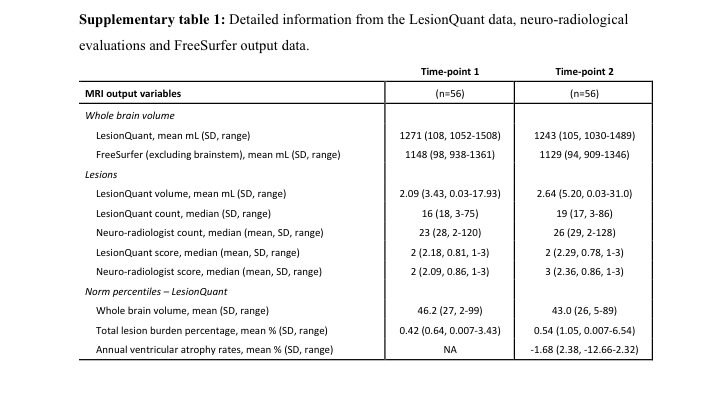

Supplement: Supplementary file 1 [file Image_1.TIFF]

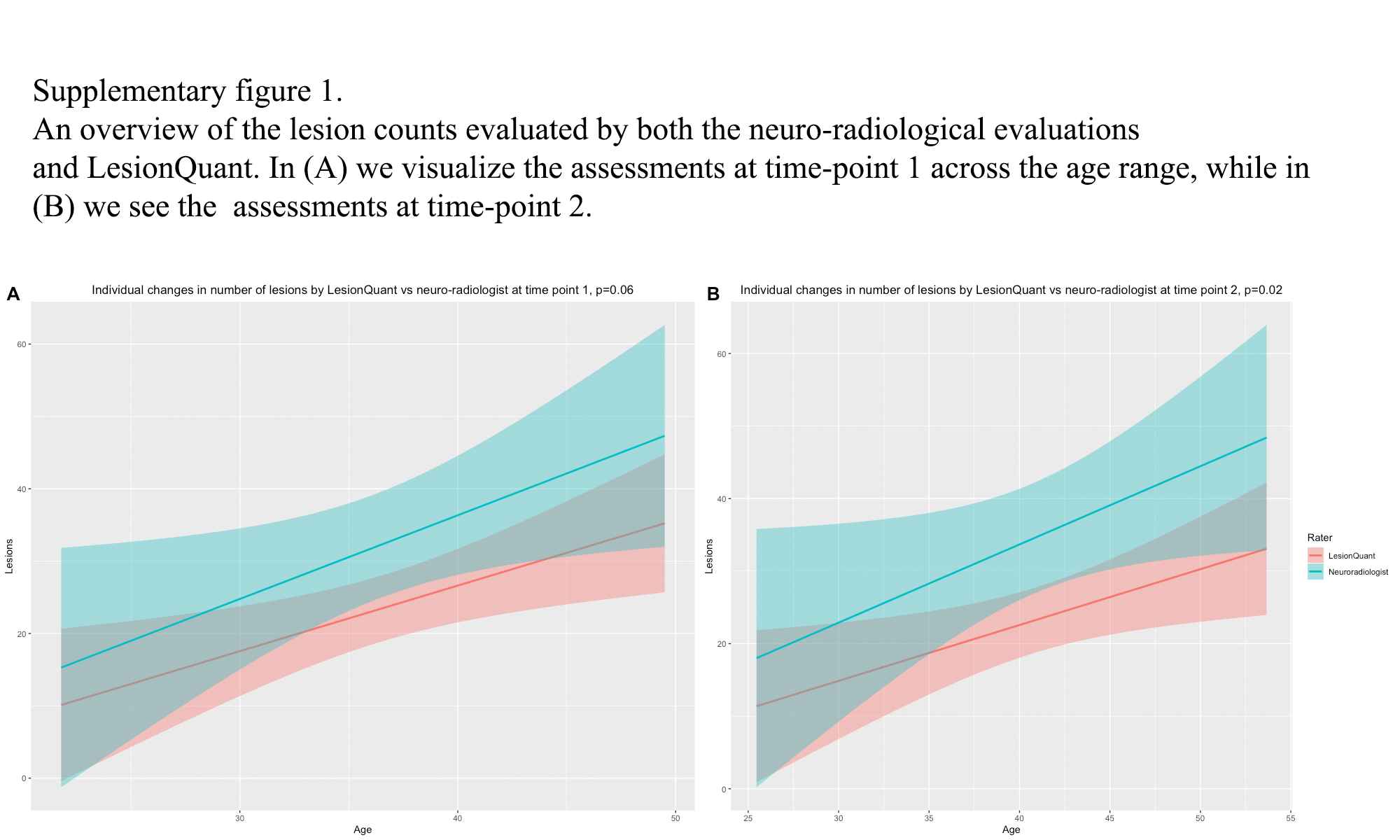

Supplement: Supplementary file 2 [file Image_2.TIFF]

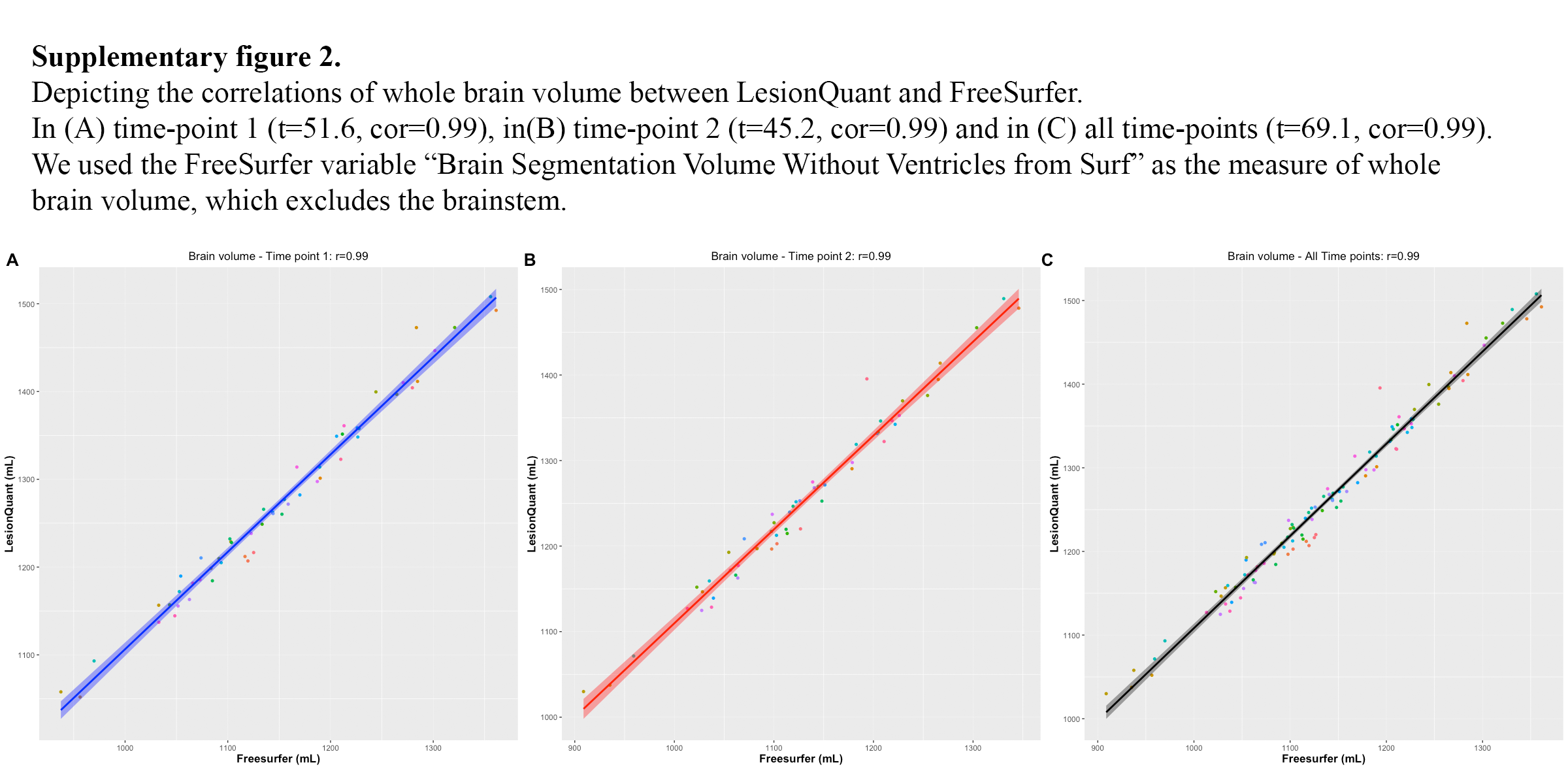

Supplement: Supplementary file 3 [file Image_3.TIFF]

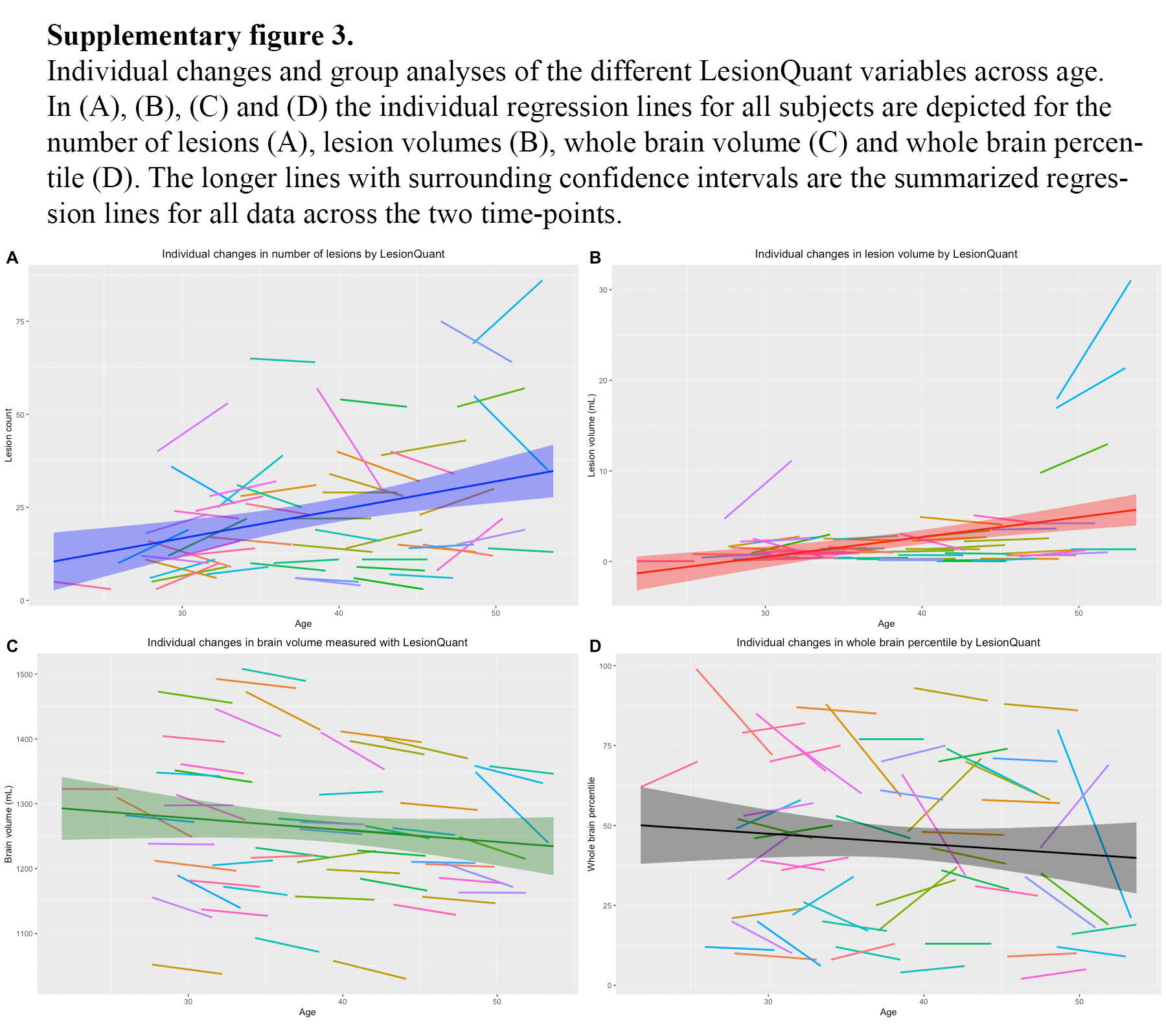

Supplement: Supplementary file 4 [file Image_4.TIFF]
